# Qual o Impacto da Evolução do Tratamento de Pacientes Portadores de Insuficiência Cardíaca na sua Mortalidade?

**DOI:** 10.36660/abc.20250329

**Published:** 2025-07-15

**Authors:** Lídia Ramalho Ribeiro Garcia, Fernanda Vitória Araújo da Silva, João Marcos Bemfica Barbosa Ferreira

**Affiliations:** 1 Universidade Nilton Lins Manaus AM Brasil Universidade Nilton Lins, Manaus, AM – Brasil; 2 Universidade do Estado do Amazonas Manaus AM Brasil Universidade do Estado do Amazonas, Manaus, AM – Brasil

**Keywords:** Insuficiência Cardíaca, Tratamento, Prognóstico

O artigo intitulado Análise de Sobrevida e Fatores Associados a Mortalidade em Portadores de Insuficiência Cardíaca na Coorte ELSA-Brasil apresenta uma significativa casuística da mortalidade em 251 pacientes com diagnóstico de insuficiência cardíaca em uma coorte brasileira de seguimento ambulatorial de longo prazo. O perfil clínico associado a indivíduos do sexo masculino, com faixa etária a partir de 70 anos, disfunção sistólica do ventrículo esquerdo e presença de comorbidades apresentou pior sobrevida. A mortalidade global após 12,3 anos de seguimento foi de 19%. Entre as classes de drogas conhecidas como redutoras de mortalidade na IC, os pacientes deste estudo utilizaram betabloqueadores, inibidores da enzima conversora de angiotensina (IECA), bloqueadores dos receptores de angiotensina (BRA) e/ou antagonistas de mineralocorticoides. Vale ressaltar também que a minoria dos pacientes (6%) apresentava fração de ejeção menor que 45%. No artigo em questão, a inclusão de pacientes no estudo foi de 2008 a 2010.^1^

Nos últimos dez anos, segundo o DATASUS, o Brasil ultrapassou 2,2 milhões de internações por insuficiência cardíaca (IC), com tendência crescente, especialmente entre 2015 e 2025. A Região Sudeste concentrou a maior parte desses casos (42,13%), seguida pelas regiões Nordeste (22,6%), Sul (22,5%), já o Norte possui uma taxa de (5,6%) e Centro-Oeste (7,5%). Observa-se uma leve predominância masculina (51,8%) e maior incidência em indivíduos com mais de 60 anos, o que reflete o envelhecimento populacional e a relação da IC com condições como hipertensão, diabetes e doença arterial coronariana.^2,3^

Os estudos BREATHE-primeira fase^4^ e BRETHE-extensão^5^ organizados pela Sociedade Brasileira de Cardiologia avaliaram o cenário de pacientes internados por IC. Na primeira fase do estudo, conduzido entre 2011 e 2012, foram avaliados 1263 pacientes com mortalidade intrahospitalar de 12,6%. Os medicamentos mais utilizados foram os betabloqueadores, IECA, BRA e antagonistas de mineralocorticoides, além de medicamentos utilizados pela descompensação como diuréticos, inotrópicos e vasodilatadores endovenosos. A fração de ejeção era preservada em cerca de 40% dos pacientes.^4^

Na segunda fase, denominada BREATHE-extensão, foram avaliados mais 1761 pacientes entre 2016 e 2018 pacientes internados por insuficiência cardíaca, totalizando um número de 3013 pacientes somando as duas fases do estudo.^5^

Entre os 3013 pacientes, a mortalidade intra-hospitalar foi de 10,9%. A mortalidade em 12 meses foi de 27,7%. Com relação a readmissão hospitalar, houve 24,3% de reinternação em 90 dias e 44,4%, em 12 meses. Os medicamentos utilizados na segunda fase foram semelhantes aos da primeira com a maioria dos pacientes utilizando betabloqueadores, IECA, BRA e antagonistas de mineralocorticoides. A utilização de sacubitril-valsartana foi iniciado durante a execução do estudo. Portanto, sua utilização antes da internação, nas primeiras 24 horas de internação, na alta, 3,6 e 12 meses após a alta, foi de 0,0%, 0,4%, 1,1%, 1,4%, 2,2% e 2,9%, respectivamente. Os inibidores da ISGLT2 ainda não eram utilizados de rotina na IC durante a realização do estudo. A fração de ejeção era maior que 50% em 28,2% dos 3013 pacientes.^5^

Após os estudos mencionados anteriormente, o tratamento para insuficiência cardíaca apresentou significativo avanço com inclusão de novas drogas tais como sacubitril-valsartana e inibidores da ISGLT. Estudos como PARADIGM-HF^6^ e DAPA-HF^7^ demonstraram significativa redução de mortalidade com uso de sacubitril-valsartana e inibidores do ISGLT-2 em pacientes com ICFER. Estas drogas têm mudado significativamente o prognóstico destes pacientes, o que impactam diretamente na mortalidade e hospitalizações. E são indicadas nas diretrizes nacionais e internacionais. Essas medicações emergem como pilares no manejo da IC atualmente, oferecendo diversos benefícios além da redução dos sintomas.^8–10^

Portanto, os estudos prévios citados acima descrevem de maneira detalhada e eficaz um número significativo de pacientes com IC, tanto ambulatoriais quanto internados, o que representa, de forma significativa, a epidemiologia da insuficiência cardíaca no Brasil. São muito importantes também como base de comparação para novos estudos que, no futuro, venham a avaliar o impacto da inclusão de novas drogas para o tratamento de IC no Brasil.
