# Supplementary material for: Qual o Impacto da Evolução do Tratamento de Pacientes Portadores de Insuficiência Cardíaca na sua Mortalidade?
Source: Arq Bras Cardiol. 2025 Jul 15;122(6):e20250329. [Article in Portuguese] doi: 10.36660/abc.20250329 (PMC13718331; doi:10.36660/abc.20250329)
Supplement: Translation

# Translation: What is the Impact of Advancements in the Treatment of Heart Failure Patients on Mortality?

**DOI:** 10.36660/abc.20250329i

**Authors:** Lídia Ramalho Ribeiro Garcia, Fernanda Vitória Araújo da Silva, João Marcos Bemfica Barbosa Ferreira

**Affiliations:** 1 Manaus AM Brazil Universidade Nilton Lins, Manaus, AM – Brazil; 2 Manaus AM Brasil Universidade do Estado do Amazonas, Manaus, AM – Brasil

**Keywords:** Heart Failure, Treatment, Prognosis

The article titled *Survival Analysis and Factors Associated with Mortality in Heart Failure Patients in the ELSA-Brasil Cohort* presents a significant case series on mortality in 251 patients diagnosed with heart failure in a Brazilian long-term outpatient follow-up cohort. The clinical profile associated with male individuals aged 70 and older, with left ventricular systolic dysfunction and the presence of comorbidities, showed poorer survival outcomes. Overall mortality after 12.3 years of follow-up was 19%. Among the classes of drugs known to reduce mortality in heart failure, patients in this study used beta-blockers, angiotensin-converting enzyme inhibitors (ACEIs), angiotensin receptor blockers (ARBs), and/or mineralocorticoid receptor antagonists. It is also worth noting that a minority of patients (6%) had an ejection fraction lower than 45%. Patient inclusion in the study occurred between 2008 and 2010.^1^

In the past ten years, according to DATASUS, Brazil has recorded over 2.2 million hospitalizations due to heart failure (HF), with an increasing trend, especially between 2015 and 2025. The Southeast region accounted for most of these cases (42.13%), followed by the Northeast (22.6%), South (22.5%), while the North and Central-West regions accounted for 5.6% and 7.5%, respectively. There is a slight male predominance (51.8%), and the highest incidence is observed in individuals over 60 years old, reflecting an aging population and the association of HF with conditions such as hypertension, diabetes, and coronary artery disease.^2,3^

The BREATHE-first phase^4^ and BREATHE-extension^5^ studies, organized by the Brazilian Society of Cardiology, evaluated the scenario of hospitalized patients with HF. In the first phase, conducted between 2011 and 2012, 1,263 patients were evaluated, with an in-hospital mortality rate of 12.6%. The most commonly used medications were beta-blockers, ACEIs, ARBs, and mineralocorticoid receptor antagonists, along with medications used for decompensation, such as diuretics, inotropes, and intravenous vasodilators. The ejection fraction was preserved in about 40% of the patients.^4^

In the second phase, called BREATHE-extension, an additional 1,761 patients hospitalized with heart failure were evaluated between 2016 and 2018, totaling 3,013 patients across both phases of the study.^5^

Among these 3,013 patients, in-hospital mortality was 10.9%, and 12-month mortality was 27.7%. Regarding hospital readmissions, 24.3% were readmitted within 90 days and 44.4% within 12 months. The medications used in the second phase were similar to those in the first, with most patients using beta-blockers, ACEIs, ARBs, and mineralocorticoid receptor antagonists. The use of sacubitril-valsartan was initiated during the study. Therefore, its use before hospitalization, within the first 24 hours of admission, at discharge, and 3, 6, and 12 months after discharge was 0.0%, 0.4%, 1.1%, 1.4%, 2.2%, and 2.9%, respectively. SGLT2 inhibitors were not yet routinely used in HF treatment during the study. The ejection fraction was above 50% in 28.2% of the 3,013 patients.^5^

Following the aforementioned studies, heart failure treatment has significantly advanced with the inclusion of new drugs such as sacubitril-valsartan and SGLT2 inhibitors. Studies such as PARADIGM-HF^6^ and DAPA-HF^7^ have demonstrated a significant reduction in mortality with the use of sacubitril-valsartan and SGLT2 inhibitors in patients with HFrEF. These drugs have significantly changed the prognosis of these patients, directly impacting mortality and hospitalizations. They are recommended in both national and international guidelines. These medications have emerged as cornerstones in the management of HF today, offering several benefits beyond symptom reduction.^8–10^

Therefore, the previous studies cited above effectively and thoroughly describe a significant number of HF patients, both outpatients and inpatients, representing the epidemiology of heart failure in Brazil. They are also very important as a comparison base for future studies that may assess the impact of introducing new drugs for HF treatment in Brazil.
